# Organic Mixed Ionic–Electronic Conductors for Organic Electrochemical Transistors: Sidechain Structure Influences Ion Uptake and Functional Performance

**DOI:** 10.1002/cphc.202500403

**Published:** 2025-09-28

**Authors:** Siyu Qin, Zeyuan Sun, Haoxuan Li, Charleen Rahman, Thomas E. Gartner, Elsa Reichmanis

**Affiliations:** ^1^ Department of Chemical and Biomolecular Engineering Lehigh University Bethlehem PA 18015 United States

**Keywords:** electrolyte interactions, operando characterization, organic electrochemical transistors, organic mixed ionic–electronic conductors, polymer semiconductors

## Abstract

Organic mixed ionic–electronic conductors (OMIECs) are an emerging class of polymeric materials with opportunities for applications in bioelectronics, neuromorphic computing, and various sensing technologies owing to their mixed conduction characteristics. The performance and long‐term operational stability of OMIECs, particularly in aqueous environments, can be influenced by the dynamic interactions between polymer functionalities and electrolyte species. This mini review highlights the necessity of integrating advanced *operando* characterization techniques and computational modeling to successfully investigate structure–property relationships. Then, recent progress in understanding how sidechain design dictates ion transport, hydration, swelling behavior, and mixed conduction properties is summarized. Furthermore, the significant impacts of electrolyte composition on doping mechanisms, structural stability, and device performance are explored; and the persistent challenges associated with extensively studied ethylene glycol sidechain designs and emerging hybrid sidechain strategies that incorporate ionic moieties are examined. Recognizing the current limitations in understanding these complex systems, particularly regarding long‐term stability, this outlook focuses on elucidating fundamental structure–property relationships and degradation mechanisms. This understanding is crucial for the rational design and future development of robust and high‐performance OMIEC materials for organic electrochemical transistor applications.

## Graphical abstract

This review explores the critical structure–property–performance relationships in organic mixed ionic–electronic conductors. By integrating advanced operando characterization and computational modeling, how polymer sidechain design and electrolyte composition dictate ion transport, ionic–electronic coupling, and electronic transport are examined. Understanding these complex interactions is key to designing next‐generation materials for bioelectronics and neuromorphic computing.

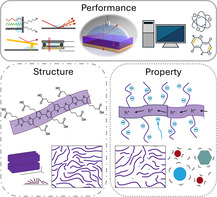

## 1 Introduction: The Emergence and Promise of OMIECs in OECTs

Organic semiconductors offer distinct advantages over traditional inorganic counterparts, including solution processability, mechanical flexibility, chemical tunability, and biocompatibility.^[^ ^1^, ^2^, ^3^ ^]^ These attributes position them as promising materials for a range of applications, such as organic light‐emitting diodes, organic photovoltaics, organic field‐effect transistors, and organic electrochemical transistors (OECTs).^[^ ^4^, ^5^, ^6^, ^7^ ^]^ The increasing demand for advanced health monitoring systems and real‐time biomarker identification has further boosted interest in organic semiconductors,^[^ ^8^, ^9^ ^]^ particularly due to their capacity for electronic signal conduction.^[^ ^8^ ^]^ Among organic semiconductors, organic mixed ionic–electronic conductors (OMIECs) represent a distinct class. Characterized by their unique ability to simultaneously transport both ions and electronic charge carriers, OMIECs are ideally suited for converting and amplifying ionic signals prevalent at biological interfaces.^[^ ^10^ ^]^ Consequently, OMIECs are highly advantageous for bioelectronic devices, electrochemical transistors, artificial synapses, ion pumps, and actuators where ionic signaling is a dominant mechanism.^[^ ^11^, ^12^ ^]^

The efficacy of OMIECs in biosensing applications critically relies on the precise control of their mixed ionic–electronic transport properties.^[^ ^13^ ^]^ Optimal biosensor performance (such as high sensitivity, selectivity, rapid response, and operational stability) is directly linked to the efficient conversion of biological ionic signals into measurable electronic outputs.^[^ ^12^ ^]^ Achieving this requires a finely tuned equilibrium between effective ion uptake from the biological sample and efficient electronic conduction within the sensor active material. For example, insufficient ion permeability can diminish sensitivity, while excessive ion uptake and swelling can degrade electronic pathways and device reliability.^[^ ^12^, ^14^ ^]^ Consequently, molecular design strategies, particularly sidechain engineering, have become paramount.^[^ ^6^ ^]^ By carefully tailoring sidechain chemical characteristics, it is possible to meticulously tune material hydrophilicity, ion affinity, and charge carrier mobility, thereby enabling the rational design of OMIECs with optimized mixed conduction for high‐performance biosensing.^[^ ^7^, ^12^ ^]^

Recent studies have explored the impact of sidechain design on how variations in sidechain chemistry/molecular structure/spacer length dictate ion transport, swelling behavior, and device performance. For instance, insertion of an alkyl spacer into the sidechain of glycolated OMIECs reduces swelling in aqueous environments.^[^ ^15^ ^]^ Moreover, these alkyl spacer‐bearing OMIECs can maintain favorable ion transport properties, leading to considerable OECT performance.^[^ ^13^, ^15^, ^16^, ^17^, ^18^ ^]^ Conjugated polymers bearing functionalized sidechains that incorporate zwitterionic groups or charged groups attached to alkyl spacers have been introduced as a means to balance hydration, swelling, and transconductance.^[^ ^13^, ^19^ ^]^ Such strategies aim to reduce excessive swelling while preserving efficient ion uptake and electronic charge carrier mobility when exposed to aqueous electrolytes.

Aside from sidechain chemistry, electrolyte properties are critical in modulating mixed conduction properties of OMIECs. Studies exploring the impact of the electrolyte highlight that the level of hydration and solvent uptake control swelling, which generally increases free volume and ion mobility within the polymer film.^[^ ^20^, ^21^ ^]^ Further, the nature of the ions (size, charge density, and solvation shell) and the electrolyte concentration affect ion transport kinetics and the efficiency of ionic–electronic coupling. These findings demonstrate that ion‐pairing interactions and water uptake significantly alter OMIEC mixed conduction properties.^[^ ^20^, ^22^ ^]^

Direct elucidation of the processes associated with ion uptake and ionic–electronic coupling under operational conditions is an imperative for analysis of the intricate relationships between polymer structure and charge transport dynamics. *Operando* methods such as grazing‐incidence wide‐angle X‐ray scattering (GIWAXS) and in situ scanning probe microscopy are crucial for capturing real‐time microstructural change. These techniques, alongside tools like electrochemical quartz crystal microbalance with dissipation monitoring (EQCM‐D) and X‐ray fluorescence analysis, allow for the direct observation of phenomena such as hydration‐induced swelling, lamellar expansion, and ion distribution. By providing insights into dynamic morphological evolution and its impact on ion mobility and electronic transport, these real‐time, multimodal approaches are indispensable for identifying structure–property–performance relationships in OMIECs.

In parallel with experimental investigations, computational modeling has become a critical approach in exploring structure–property relationships of OMIECs from the atomic to the molecular scale. In particular, electronic structure methods and/or molecular simulations have provided insight into how chemical structure, electrolyte identity, and dynamic morphological responses dictate mixed conduction behavior.^[^ ^23^, ^24^, ^25^ ^]^ These computational approaches offer a complementary view to *operando* characterization, enabling mechanistic understanding of electrolyte–polymer interactions, charge localization, and transport asymmetries that are challenging to resolve experimentally.^[^ ^26^, ^27^ ^]^

Despite significant progress in investigating OMIEC structure–property–performance relationships, considerable research challenges remain. A primary concern revolves around understanding and improving the long‐term operational stability of OMIECs under various electrolyte environments and during repeated electrochemical (de)doping cycles. Furthermore, the intricate interplay between molecular design, thin‐film microstructure, and resulting mixed ionic–electronic conduction makes it difficult to establish clear predictive models. This review underscores the growing effort to establish comprehensive OMIEC sidechain design principles that promote efficient ion transport, along with specificity for and sensitivity to various ion types. Systematic correlation of electrolyte interactions, hydration behavior, and charge transport mechanisms will contribute to the development of high‐performing OMIEC materials for bioelectronic and electrochemical applications.

## 2 Mechanisms of Mixed Ionic–Electronic Conduction

The hallmark of OMIECs is their ability to conduct both ions and electronic charge carriers. The mixed conduction properties arise from a complex interplay between molecular structure, molecular packing, and interactions with the surrounding electrolyte environment. In OMIECs, electronic and ionic transport occur in distinct yet tightly coupled pathways.^[^ ^6^, ^28^ ^]^ Electronic charge transport primarily takes place along the conjugated polymer backbone (intrachain transport) and *via* hopping between adjacent chains or conjugated segments (interchain transport).^[^ ^28^, ^29^ ^]^ The efficiency of this process is highly dependent on backbone planarity, *π*–*π* interactions, and the presence of crystalline and/or ordered domains.^[^ ^17^, ^30^ ^]^ Simultaneously, ion transport is understood to mostly proceed through amorphous regions,^[^ ^31^ ^]^ which facilitate ion permeation due to their inherently disordered molecular packing and presence of polar or ion coordinating sidechains.^[^ ^11^ ^]^ Alternatively, in blended ion‐conducting polymers such as poly(3,4‐ethylenedioxythiophene):polystyrene sulfonate (PEDOT:PSS), these transport pathways can exist within the ion‐conducting polymer domains (e.g., polystyrene sulfonate (PSS)).^[^ ^11^, ^17^, ^32^ ^]^ In both scenarios, the sidechains and ion‐conducting domains promote ion solvation.^[^ ^31^, ^32^, ^33^ ^]^ As a result, upon contact with the electrolyte, ions can permeate through amorphous regions by transferring between coordination sites, often assisted by the segmental motion of the polymer chains or by diffusion within the absorbed electrolyte phase.^[^ ^28^ ^]^

**Figure 1:**
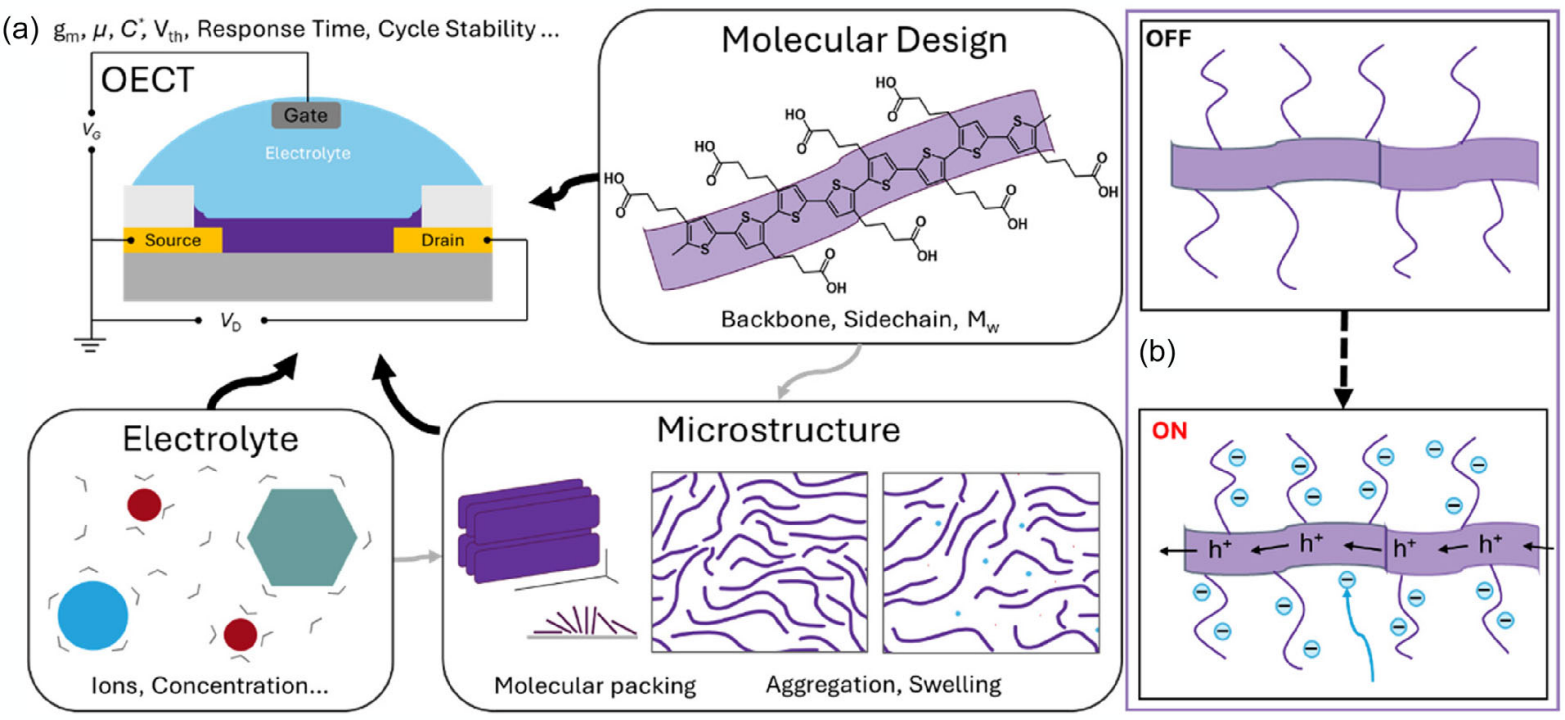
a) The OECT archtecture and the interplay of OMIEC molecular design, electrolyte properties, and thin‐film microstructure in the ionic–electronic mixed conduction properties of the channel material in OECT. b) Schematic illustration of electrochemical doping mechanism of an accumulation‐mode p‐channel OECT (e.g., with P3HT as channel material).

Ionic–electronic coupling can occur when ions penetrate into the OMIEC channel material.^[^ ^28^, ^34^ ^]^ Upon application of a doping potential through the gate electrode of the OECT (**Figure** **1**a), ions from the electrolyte are injected into the bulk of the conjugated polymer (the channel material), and, as a consequence, form polarons.^[^ ^35^ ^]^ Polarons are believed to stabilize the nearby electronic charge carriers, facilitating electronic charge transport.^[^ ^34^ ^]^ For instance, in a well‐studied accumulation mode p‐channel material, poly(3‐hexylthiophene‐2,5‐diyl) (P3HT), oxidation of the backbone (injection of holes) is accompanied by the injection of anions into the polymer matrix when a doping potential is applied through the gate electrode (Figure 1b). Thus, the electrochemical doping process directly modulates the concentration and mobility of electronic charge carriers, thereby modulating electronic conductivity of the channel material.

In addition to molecular design, there are several other factors that can influence mixed conduction properties. These factors include: 1) the identity of the ions (size, charge density, and solvation shell) and the amount of ion uptake, which are particularly important for mixed conduction;^[^ ^20^, ^21^ ^]^ 2) the amount of water uptake and electrolyte concentration that profoundly affect ion transport kinetics and ionic–electronic coupling; and 3) thin‐film microstructure (degree of crystallinity and the extent of phase separation in blended or block copolymer systems).^[^ ^28^, ^36^, ^37^, ^38^ ^]^ Therefore, a detailed review of how sidechain design and electrolyte composition affect these aforementioned factors and further alter the mixed conduction process and OECT performance follows.

## 3 Benchmarking OECT Performance: Figure of Merit

Before examining the details of the impact of sidechain design and electrolyte composition on the mixed conduction process, it is important to provide a series of well‐defined parameters which can quantify the material's intrinsic capabilities for mixed conduction.^[^ ^39^, ^40^ ^]^ Thus, this figure of merit for OECTs is established as the product of volumetric capacitance (C*) and electronic charge carrier mobility (*μ*).

(1) $$g_{m}=\frac{Wd}{L}\mu C^{*}\left(V_{\text{th}}-V_{G}\right)$$

In the equation benchmarking OECT performance (Equation 1), *g*_m_ is the OECT transconductance, *Wd/L* is the geometry factor of the channel in given OECT configuration, (*V*_th_ *− V*_G_) is the gate bias offset, *μ* is the electronic carrier mobility, and C* is the volumetric capacitance.

Volumetric capacitance (C*) quantifies the charge storage capacity of the OMIEC material per unit volume and represents the change in the number of stored ionic charge carriers per unit volume of the OMIEC in response to a small change in electrochemical doping potential.^[^ ^40^ ^]^ C* is typically measured via electrochemical impedance spectroscopy (EIS) in either a three‐electrode setup or an OECT configuration.^[^ ^35^, ^41^ ^]^ A high C* suggests that the material can efficiently uptake, transport, and store a large number of ions throughout its bulk when gate voltage is applied. Thus, C* directly reflects the OMIEC ability to participate in the volumetric electrochemical doping process that underpins OECT functionality. Effective OMIECs generally exhibit C* values in the range of 100–300 F*cm^−3^. Distinct C* values are reported for several notable conjugated polymers: for instance, the well‐known p‐channel conjugated OMIEC material poly[2‐(3,3′‐bis(2‐(2‐(2‐methoxyethoxy)ethoxy)ethoxy)‐[2,2'‐bithiophen]‐5‐yl)thieno[3,2‐b]thiophene], commonly abbreviated as p(g2T‐TT), demonstrated a C* of ≈240 F* cm^−3^.^[^ ^42^ ^]^ C* values of ≈242 F*cm^−3^ and 160 F*cm^−3^ were documented for the glycolated polythiophenes poly(3‐[2‐[2‐(2‐methoxyethoxy)ethoxy]ethyl]thiophene‐2,5‐diyl) (P3MEEET)^[^ ^15^ ^]^ and poly(3‐[2‐[2‐(2‐methoxyethoxy)ethoxy]methyl]thiophene‐2,5‐diyl) (P3MEEMT), respectively.^[^ ^15^ ^]^ Furthermore, a C* of 111 F*cm^−3^ was reported for the representative carboxylic acid polythiophene poly[3‐(4‐carboxybutyl)thiophene‐2,5‐diyl] (P3CBT).^[^ ^43^ ^]^ Additionally, a high C* of 589 F*cm^−3^ was recorded for the extensively studied n‐channel conjugated polymer poly(benzobisimidazobenzophenanthroline) (BBL).^[^ ^44^ ^]^

*Electronic charge carrier mobility* (*μ*) is defined as the velocity of electronic charge carriers per unit electric field within the OMIEC^[^ ^40^ ^]^ and is a measure of how efficiently electronic charges can move through the channel.^[^ ^28^ ^]^ Higher mobility contributes to a larger drain current, whereby high charge carrier mobility is crucial for achieving fast OECT response time for turn‐on and turn‐off. For many p‐channel OMIECs operating in aqueous electrolytes, hole mobilities typically range from 0.1 to 5 cm^2^ V^−1^ s^−1^. For instance, a *μ* of 0.05 cm^2^ V^−1^ s^−1^ was noted for P3MEEET,^[^ ^15^ ^]^ and P3MEEMT was characterized by a *μ* of 0.23 cm^2^ V^−1^ s^−1^.^[^ ^15^ ^]^ The difference in hole mobility between these two polymers (P3MEEET and P3MEEMT) can be attributed to the nature of the spacer linking the ethylene glycol sidechain to the polythiophene backbone. In P3MEEMT, the shorter methyl spacer leads to a more planar backbone and stronger interchain interactions, resulting in a higher charge mobility compared to P3MEEET, which has an ethyl spacer. In contrast, a higher *μ* of ≈0.96 cm^2^ V^−1^ s^−1^ was reported for P3CBT.^[^ ^43^ ^]^ However, even greater hole mobilities are found in some glycolated polythiophenes with different backbone molecular structure. As an example, poly(2‐(4,4′‐bis(2‐methoxyethoxy)‐5′‐methyl‐[2,2′‐bithiophen]‐5‐yl)‐5‐methylthieno[3,2‐b]thiophene) (PgBTTT) exhibits a *μ* of 3.44 ± 0.13 cm^2^ V^−1^ s^−1^;^[^ ^42^ ^]^ and a high‐performance variant of p(g2T‐TT), p(g_4_2T‐TT) demonstrated a hole mobility of 6.53 ± 0.07 cm^2^ V^−1^ s^−1^.^[^ ^45^ ^]^ In n‐channel OMIECs, electron mobilities are generally lower than hole mobilities in p‐channel counterparts. For instance, the reported BBL electron mobility (*μ*) is 4.4 × 10^−2^ cm^2^ V^−1^ s^−1^.^[^ ^44^ ^]^

The product of *μ* and C* (μC*) is the accepted *figure of merit* for OMIEC performance in an OECT configuration and is widely used for benchmarking to allow for direct performance comparisons that are independent of device geometry.^[^ ^39^ ^]^ A higher μC* indicates a greater ability to modulate OMIEC electronic conductivity in response to changes in ion concentration driven by the gate voltage.^[^ ^39^, ^46^ ^]^ Enhanced modulation can translate to higher transconductance and thus greater signal amplification. The development of OMIECs has been marked by a steady rise in their *figure of merit*, μC*. Since 2014, this *figure of merit* has increased by three orders of magnitude, with numerous high‐performing OMIECs achieving μC* in the range of several hundred F*cm^−1^  V^−1^ s^−1^. For example, PgBTTT reports a μC* of 502 ± 18 F*cm^−1^  V^−1^ s^−1^.^[^ ^42^ ^]^ Subsequently, a significant benchmark was set by P(g_4_2T‐TT), which, through meticulous synthetic purification and processing, attained a record μC* of ≈(2.0 ± 0.1) × 10^3^ F*cm^−1^  V^−1^ s^−1^, equivalent to ≈2,000 F*cm^−1^  V^−1^ s^−1^.^[^ ^45^ ^]^

## 4 Operando Characterization Techniques as Tools for Structure–Property–Performance Analysis

**Figure 2:**
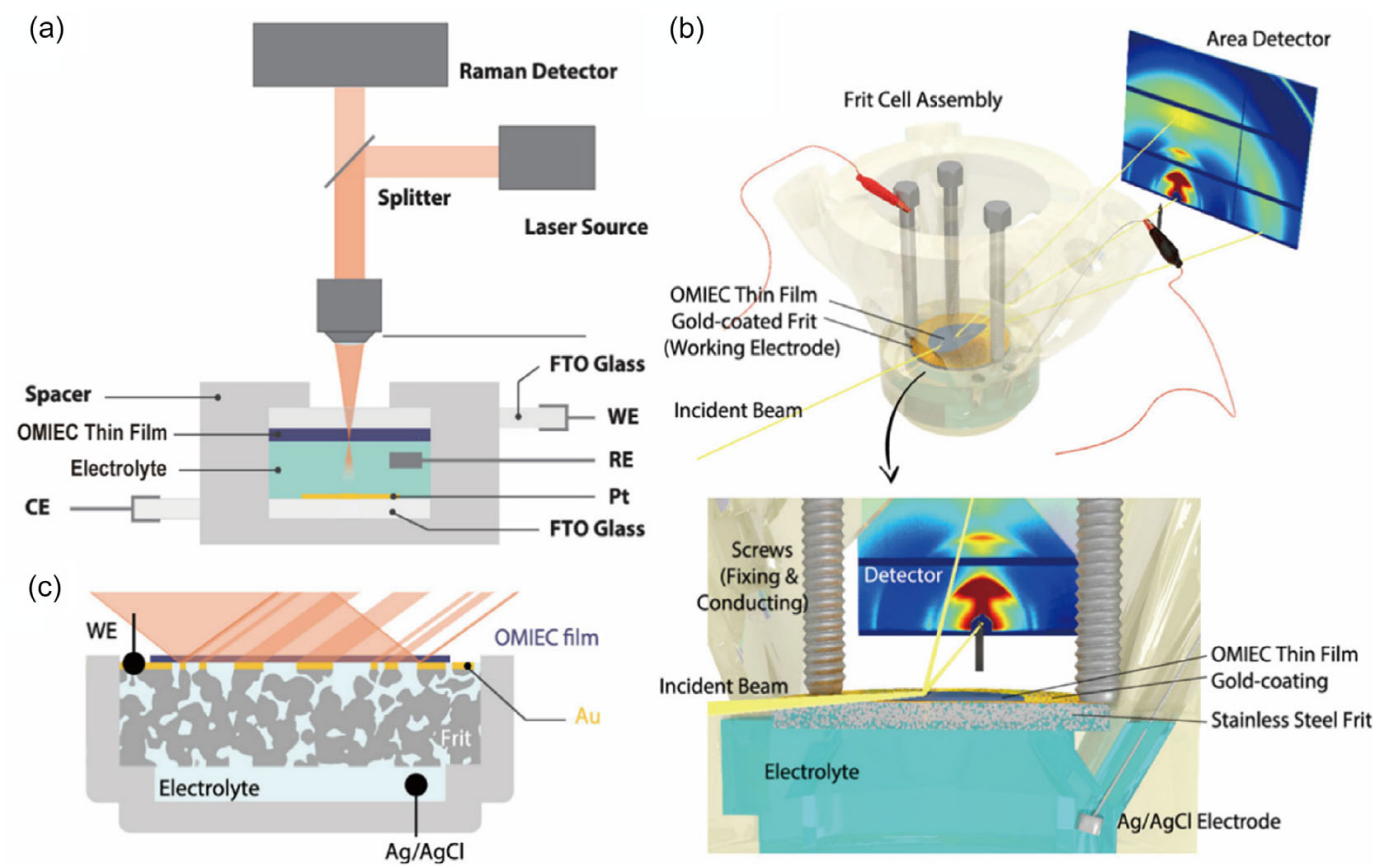
a) A typical *operando* Raman cell in which the sample is held upside down on the electrolyte. b) 3D rendering of the printed *operando* cell with porous frit substrate/working electrode, highlighting the underlying electrolyte reservoir and electrolyte path coupling the film of interest to potentiostatic control. c) The side view of the frit cell. Reproduced under terms of the CC BY‐NC‐ND 4.0 license.^[^ ^34^ ^]^ Copyright 2022, The Authors, American Chemical Society.

Several advanced characterization techniques have emerged as critical tools to evaluate OMIEC structure–performance relationships. In particular, *operando* Raman spectroscopy (**Figure** **2**a), *operando* GIWAXS (Figure 2b,c), in situ atomic force microscopy (AFM), and AFM‐based scanning probe techniques allow for correlation of polymer structure and ion transport dynamics under operational conditions.^[^ ^34^, ^47^, ^48^, ^49^ ^]^

For instance, *operando* Raman has been used to capture real‐time changes in the vibrational signatures of neutral/charged species,^[^ ^43^, ^50^ ^]^ while *operando* GIWAXS effectively provides insight into changes in polymer film microstructure that results from lamellar expansion,^[^ ^51^ ^]^ and correlation of charge carrier density with microstructure changes.^[^ ^52^ ^]^ Combined, the data correlate structural changes (molecular packing and change in packing distance) to the dynamic evolution of chemical species and their associated charge states within the polymer film during operation.^[^ ^47^ ^]^

In situ grazing incidence X‐ray fluorescence (GIXRF) analysis provides real‐time elemental analysis, revealing how electrolyte exposure and electrochemical cycling manipulate mesoscale ion distribution.^[^ ^53^, ^54^ ^]^ The combined application of GIWAXS and GIXRF enables the direct observation of the dynamic structural and compositional changes within OMIEC thin films during operation. The immediate feedback provided by these correlated measurements is vital for unequivocally establishing the relationship between transient microstructural/compositional evolution events and their instantaneous impact on OECT performance metrics, including electronic charge transport.

In situ AFM, operated within aqueous environments, enables real‐time qualification of polymer film thickness modulations and swelling dynamics during processes such as electrochemical doping.^[^ ^52^ ^]^ This capability allows for direct observation of solvent uptake and structural transformations under operational conditions. A significant advancement, photoinduced force microscopy (PiFM), integrates AFM with tunable infrared laser sources to achieve nanoscale chemical imaging concurrently with surface topography acquisition. By tuning the laser to specific vibrational frequencies, PiFM can effectively map the distribution of dopant ions or distinct chemical states within polymer films.^[^ ^38^, ^52^, ^55^, ^56^ ^]^ Furthermore, AFM‐based scanning probe methodologies are increasingly utilized to interrogate local nanomechanical properties, including elastic modulus, adhesion forces, and viscoelastic behavior.^[^ ^57^ ^]^ *Operando* electrochemical AFM, for example, can elucidate the evolution of these mechanical properties under electrochemical stimuli, thereby correlating ion uptake and hydration phenomena with changes in material compliance, such as film plasticization or rigidification.^[^ ^58^ ^]^ Such analyses are critical for elucidating the complex interplay between electrochemical processes and mechanical responses of functional polymer films.

From a gravimetric perspective, integrating the aforementioned *multimodal* techniques with real‐time mass change measurements is instrumental for uncovering fundamental behavior related to ion uptake, which is a critical process for OMIEC channel materials operated in OECT configuration. For example, Sun et al. demonstrated a combination of *operando* GIXRF along with EQCM‐D, directly revealing that noncompensating ions strongly interact with carboxylated polythiophene. This interaction drives a deswelling‐doping behavior due to strong cation expulsion during anion injection, revealing the ion–polymer interaction mechanism in conjugated polyelectrolyte categories.^[^ ^53^ ^]^ Additionally, Tropp et al. demonstrated a combination of *operando* GIWAXS with EQCM‐D, where they elucidated that the choice of electrolyte dictates the dominant doping mechanism in P3MEEET and its interplay with polymer molecular weight.^[^ ^59^ ^]^ Specifically, in NaCl electrolyte, doping proceeded *via* anion (Cl^−^) uptake, causing lamellar expansion and an increase in film mass and dissipation. Conversely, when potassium bis(trifluoromethylsulfonyl)imide electrolyte was used, doping occurred primarily through cation (K^+^) expulsion. This led to a decrease in film mass, a contraction in lamellar spacing, and reduced dissipation, indicative of a more densified polymer structure that enhanced charge percolation pathways. Furthermore, Savva and coworkers employed EQCM‐D in conjunction with EIS to quantitatively assess how systematically increasing the fraction of hydrophilic ethylene glycol units presenting in the sidechains influenced mass uptake and OECT device performance.^[^ ^60^ ^]^ Their findings indicated that while the degree of swelling and C* initially correlated with increasing ethylene glycol content, the polymer variant with the highest hydrophilicity demonstrated the most significant mass uptake yet a diminished C*. This observation suggests a trade‐off, wherein excessive solvent uptake can compromise efficient ion uptake and charge storage capacity. Thus, the integration of real‐time mass monitoring with other characterization methods is utilized to reveal complex ion–polymer interaction mechanisms, elucidate the influence of electrolytes on doping processes, and assess how polymer structure impacts mass uptake and device performance.

Simulations can further clarify how structural dynamics influence charge transport properties in OMIECs. As an example, molecular dynamics (MD) simulations combined with quantum chemical calculations revealed that ion distribution and structural rearrangements in hydrated p(g2T‐T) give rise to temporal fluctuations in highest occupied molecular orbital (HOMO) energy levels on the picosecond timescale.^[^ ^61^ ^]^ A recent review of density functional theory (DFT) and MD approaches highlighted their complementary roles in modeling orbital energetics, doping mechanisms, and swelling behavior in OMIECs, emphasizing their role in facilitating interpretation of data from *operando* measurements.^[^ ^34^ ^]^ Other computational efforts have shown that DFT captures effects missed by classical models, such as the formation and energetics of polarons and bipolarons, the description of oxidation states and charge localization, and the redox processes relevant to electrochemical doping.^[^ ^62^ ^]^

Together, these techniques offer a comprehensive view of how ionic sidechains mediate ionic and electronic transport through dynamic morphological evolution.

## 5 Sidechain Design and Mixed Conduction

**Figure 3:**
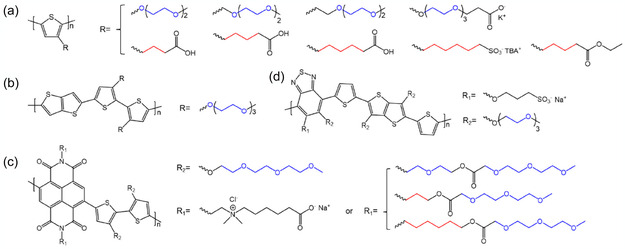
Representative examples of OMIEC chemical structures: a) polythiophene‐based polymers bearing glycolated and ionic sidechains. b) p(g2T‐TT). c) NDI‐T2‐based conjugated polymers with glycolated and zwitterionic sidechains. d) PgBT(Ion)2gTT conjugated polymer with both ionic and glycolated sidechains.

The mixed conduction performance of conjugated polymers depends on both backbone molecular architecture and sidechain molecular structure. While several recent reviews focus on alternative backbone chemistries,^[^ ^17^, ^63^, ^64^, ^65^ ^]^ the influence of sidechain design on OMIEC characteristics has been less well studied even though synthetic modifications such as incorporation of ethylene glycol units or ionic moieties significantly dictate swelling and ion uptake.^[^ ^15^, ^43^, ^66^, ^67^ ^]^ Note, the sidechain directly impacts ion mobility, electronic conductivity, and device stability.^[^ ^13^, ^16^, ^17^, ^21^, ^47^, ^53^ ^]^ **Figure** **3** provides a summary of molecular structures that have been applied to OMIEC designs, including electron‐rich and electron‐poor conjugated units required for electronic charge transport, and a range of alternative sidechain chemistries that promote polymer–electrolyte interactions and ion transport.

For instance, sidechains containing ethylene glycol units, which may include alkyl spacers of various lengths (Figure 3a,b), are prominent in the design of OMIECs owing to several advantages.^[^ ^17^ ^]^ *First*, the ether oxygen atoms presenting in the glycolated sidechain can effectively coordinate with and solvate a wide array of ionic species, significantly enhancing ion mobility within the polymer matrix.^[^ ^17^, ^68^ ^]^ As a result, rapid ion insertion and migration are facilitated, leading faster doping kinetics compared with polythiophenes bearing alkyl sidechains (e.g., P3HT).^[^ ^38^ ^]^ Furthermore, doping kinetics can be further accelerated by inserting additional oxygen atoms into the sidechains or positioning the oxygen elements further away from the polythiophene backbone.^[^ ^69^ ^]^

### 5.1 Glycolated Sidechains: The Double‐Edged Sword of Hydrophilicity

*Second*, the hydrophilic nature of ethylene glycol‐containing sidechains can promote water and ion uptake.^[^ ^13^, ^15^, ^37^ ^]^ In one case of n‐channel naphthalene tetracarboxylic diimide and bithiophene‐based copolymers (NDI‐T2), incorporation of ethylene glycol sidechains led to higher C* in comparison to branched alkyl side chain designs.^[^ ^68^ ^]^ However, in another case based on a series of p(g2T‐TT) variants, a trade‐off is suggested by the results, wherein efficient ion uptake and C* can be compromised by excessive water uptake.^[^ ^60^ ^]^ Besides, the interconnectivity between adjacent polymeric crystalline domains can be disrupted by excessive swelling.^[^ ^38^ ^]^ Consequently, *μ* may be reduced. Such swelling can also induce substantial and uneven volumetric changes between crystalline and amorphous regions.^[^ ^38^ ^]^ The long‐term operational stability and overall OECT performance of devices are, in turn, negatively impacted by these heterogeneous volumetric changes.^[^ ^15^, ^17^, ^21^, ^38^, ^47^, ^70^, ^71^, ^72^, ^73^ ^]^

*Third*, the incorporation of hydrophilic ethylene glycol sidechains not only facilitates enhanced ion transport and promotes mass uptake within the OMIEC material, but it also gives rise to a noteworthy phenomenon in the presence of diverse cationic and anionic species within the electrolyte. Conventionally, the doping mechanism in p‐channel OMIEC materials is predominantly hypothesized to involve solely anion and concomitant solvent uptake. However, in specific instances involving P3MEEMT and P3MEEET, two typical p‐channel glycolated polythiophenes characterized by excessive hydrophilicity, cations may be incorporated during the passive swelling.^[^ ^38^, ^59^ ^]^ The results suggest a competitive interplay between cation repulsion and anion injection during the electrochemical doping process. Thus, further studies are required to fully elucidate the underlying mechanisms of this interplay and to identify critical parameters for controlling the degree of passive cation uptake. These investigations present significant opportunities to devise strategies for precisely managing polymer swelling, thereby enabling the creation of OMIEC materials with enhanced performance and operational stability.

MD simulations provide added molecular‐level insights into the role of sidechain chemistry in OMIECs and underscore the crucial role of sidechains in controlling hydration behavior, segmental mobility, and morphological evolution. For example, coarse‐grained MD simulations using the Martini force field showed that the protonation state of PSS dictates phase separation in PEDOT:PSS, with micelle‐like domains forming at both low and high pH conditions and more homogeneous mixing formation at intermediate pH, altering water distribution and solvent‐accessible surface area.^[^ ^23^ ^]^ Simulations of oxidation‐induced swelling in [2,5‐bis(thiophenyl)‐1,4‐bis(2‐(2‐(2‐methoxyethoxy)ethoxy)‐ethoxy)benzene] (PB2T‐TEG) systems revealed water and ion uptake lead to lamellar expansion. But these transitions are significantly modulated by substrate interactions, with *π*–*π* stacking reemerging at higher oxidation levels.^[^ ^25^ ^]^ In a systematic investigation of sidechain polarity, coarse‐grained MD combined with kinetic Monte Carlo simulations was used to study how varying the fraction of glycolated (polar) versus alkyl (apolar) sidechains in a polythiophene‐based OMIEC affects electrolyte percolation, morphology, and charge mobility.^[^ ^74^ ^]^ Radial distribution functions revealed electrolyte localization near polar sidechains, and charge transport was maximized at 40% and 100% polar sidechain fractions, while intermediate compositions disrupted *π*–*π* organization and reduced polaron delocalization.

In conclusion, while incorporating ethylene glycol sidechains offers benefits like enhanced doping kinetics and increased ion uptake, potentially leading to a high volumetric capacitance C*, a critical trade‐off exists. These same sidechains can compromise electronic conduction by lowering *μ* if the overall molecular design isn't carefully optimized.

### 5.2 The Role of Alkyl Spacers in Modulating Morphology and Swelling

A popular way of carefully tuning the performance trade‐off between *μ* and C* (ion uptake) is through adjusting the sidechain alkyl spacer length. Longer alkyl spacers reduce degree of swelling and improve mixed conduction by maintaining thin‐film morphology.^[^ ^16^, ^43^ ^]^ In particular, Maria et al. showed that incorporating propyl or hexyl spacer units between the conjugated polymer backbone and the ethylene glycol moiety in n‐channel NDI‐T2‐based copolymers (Figure 3c) effectively suppressed water‐induced swelling during electrochemical (de)doping processes in aqueous electrolytes.^[^ ^16^ ^]^ This structural modification preserved lamellar packing distance and prevented the disruption of *π*–*π* stacking interactions. These effects enhanced electronic transport, leading to improved *μ* and cycle stability in OECT devices. A hybrid all‐atom/united‐atom MD approach demonstrated that adding a methylene spacer into the sidechains of polythiophene enhanced sidechain flexibility and Li^+^ mobility in amorphous domains, but this could also lead to the formation of ion trapping within crystalline domains due to tighter packing.^[^ ^24^ ^]^ Complementary findings demonstrated that in the carboxylated polythiophene family, extending the length of alkyl spacer between the conjugated backbone and the terminal carboxylic acid functional group reduced the degree of swelling while maintaining moderate OECT performance.^[^ ^43^ ^]^ In situ Raman spectroscopy and spectroelectrochemical measurements revealed that these spacer‐tuned sidechains facilitated efficient ion uptake without compromising the structural integrity of the polymer films. In combination, the results highlight the importance of *spacer length optimization* in balancing hydration control, ion uptake, and morphological robustness for design of high‐performance OMIEC materials.

### 5.3 Ionic and Hybrid Sidechains: Advanced Strategies for Decoupling Hydration and Ion Transport

Furthermore, the widespread adoption of ethylene glycol sidechains, due to their recognized advantages, may also limit the exploration of other promising sidechain types. Ionic sidechains, for example, could offer unique and beneficial characteristics when integrated with various polymer backbones, leading to novel channel materials for diverse OECT applications. For example, polythiophenes with carboxylic‐acid‐bearing sidechains (Figure 3a) exhibit somewhat lower C* in comparison to their glycolated analogs, and they undergo deswelling during^[^ ^53^ ^]^ doping, which implies that cation repulsion dominates the mass exchange along electrochemical doping process.^[^ ^53^ ^]^ In contrast, ester‐functionalized analogs undergo anion uptake‐dominated swelling as observed among many p‐channel polythiophenes.^[^ ^53^ ^]^

Additionally, incorporation of hybrid sidechain architectures such as those that combine a spacer unit (alkyl or ethylene glycol) with ionic moieties or featuring zwitterionic groups is another strategy to optimize ion uptake and control polymer swelling. The approach mitigates excessive swelling that can disrupt the structural integrity of the polymer film and compromise electronic transport, thereby enhancing device performance and cycle stability in aqueous environments.^[^ ^13^, ^19^ ^]^ In addition, sulfonate (—SO_3_^−^) end groups promote hydrophilicity and enable efficient ion penetration from the aqueous electrolyte into the bulk polythiophene (Figure 3c).^[^ ^66^ ^]^ Yet another approach uses sidechains comprising both ethylene glycol units and potassium carboxylate moieties to produce the water‐soluble conjugated polyelectrolyte, poly(potassium 3‐(2‐(2‐(2‐(thiophen‐3‐yloxy)ethoxy)ethoxy)ethoxy)propanoate)‐2,5‐thiophene‐diyl) (PCAT‐K) (Figure 3a). Subsequent mixing of PCAT‐K with BBL formed the water‐processable BBL:PCAT‐K blend, which exhibits n‐channel semiconducting behavior and vastly increased electronic conductivity compared to pristine BBL.^[^ ^75^ ^]^ Similarly, Ding et al. demonstrated a postpolymerization method to introduce anionic sidechains onto donor–acceptor conjugated polymers (Figure 3d) that also led to enhanced C* and *μ* compared to fully glycolated counterparts.^[^ ^19^ ^]^ For conjugated polymers with zwitterionic sidechains (Figure 3c), intrinsic charge neutrality of the sidechains enabled ionic conduction without inducing significant volumetric change.^[^ ^13^, ^76^ ^]^ Such sidechains preserved electrochemical reversibility, increased redox stability, and enhanced mixed conduction performance in OECTs operating in physiological saline environments.^[^ ^13^ ^]^ The hybrid sidechains promoted efficient ion exchange while suppressing excessive water uptake, resulting in improved crystallinity and reduced morphological deformation during device operation. Broadly, the highlighted studies show that integrating ionic functionalities into a single sidechain provides a strategy to decouple hydration from destabilizing swelling, yielding materials that perform more reliably in aqueous electrolyte systems.

Collectively, the results obtained from investigations involving glycolated and various ionic sidechains strongly indicate that sidechain functionality is a primary determinant of electrochemical doping processes and key performance metrics. Additionally, these case analyses validate sidechain engineering as a critical approach for elucidating structure–property–performance relationships and for guiding the further optimization of OMIEC properties.

## 6 Electrolyte Effects and Charge Transport

### 6.1 The Role of Ions and Solvent

Aside from sidechain molecular design, the interaction between electrolytes and sidechains has a profound influence on OMIEC structure and performance. For instance, Yu et al. demonstrated that an n‐channel perylene diimide‐based OMIEC functionalized with crown ether sidechains exhibited highly selective responses depending on the particular aqueous electrolyte cation, with K^+^ enabling significantly higher source–drain (S–D) current than sodium ions (Na^+^).^[^ ^77^ ^]^ This effect was attributed to favorable interactions of K^+^ with the oligoether sidechains, enhancing charge carrier transport via a “sandwich‐like” molecular aggregate structure. Selective complexation promoted molecular aggregation and improved electron transport, illustrating how ion identity can directly modulate both structure and device performance. Similarly, Sun et al. found that p‐channel carboxylic acid‐functionalized polythiophene mixed conduction polymers displayed a special doping behavior in which the mass uptake process is governed by cation expulsion resulting in mass decrease during doping.^[^ ^53^ ^]^ These studies underscore the complex and ion‐specific interactions between sidechains and electrolyte species that control OMIEC performance and structural integrity in aqueous environments.

**Figure 4:**
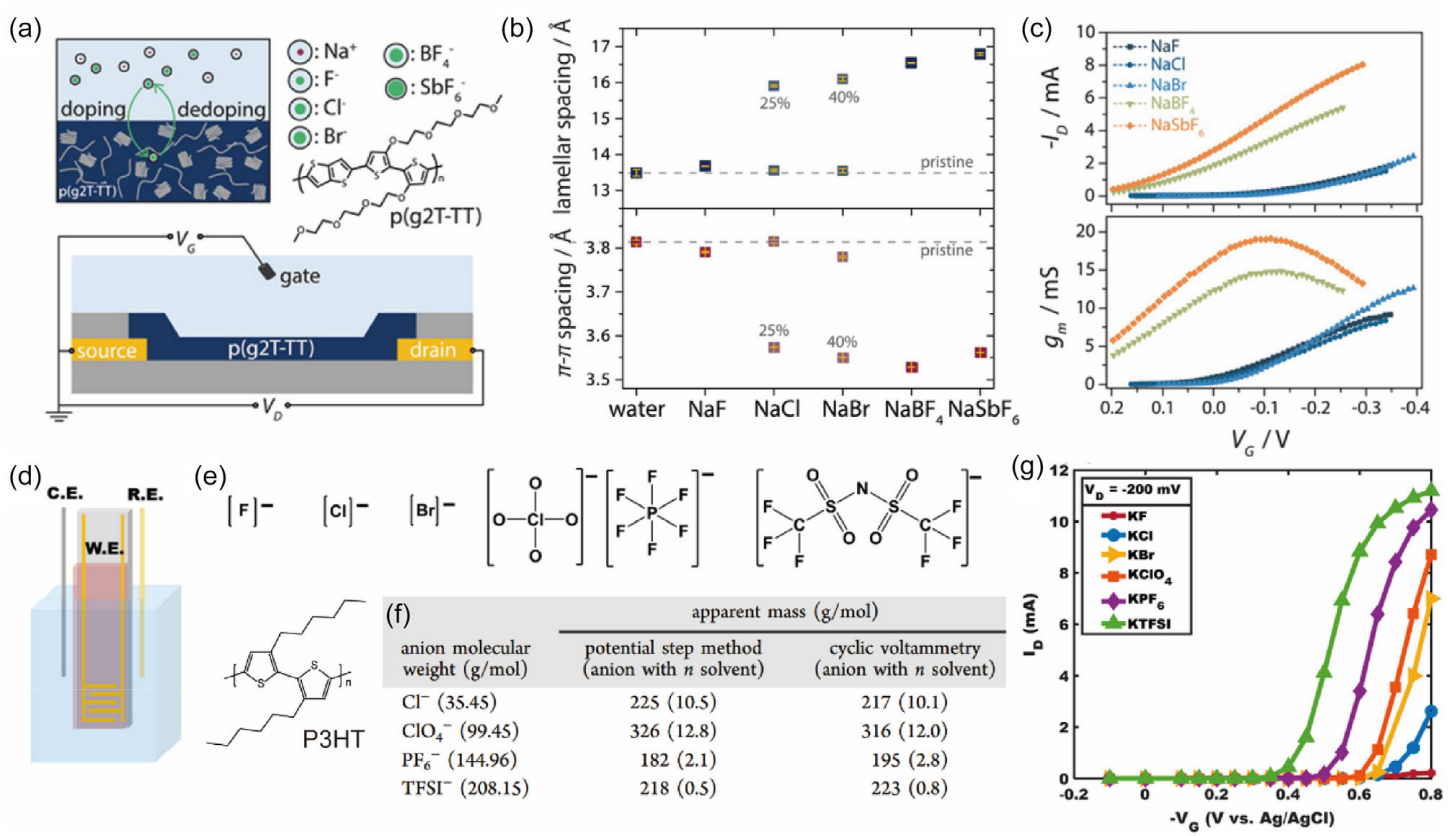
a) Molecular structure of p(g2T‐TT) and schematic of the interactions with ions at the film level (top) during OECT (bottom) operation. Cations are shown in red and the various anions used in this study in green. A representative hydration shell surrounding the ions (in free electrolyte) is indicated in light blue. b) Statistically predominant lamellar (top) and *π*–*π* stacking distances (bottom) extracted from fitting the diffraction peak positions. c) Transfer characteristics (top) and transconductance (bottom) of identical OECTs gated with various electrolytes at V_D_ = −0.4 V. Reproduced with permission.^[^ ^22^ ^]^ Copyright 2018, WILEY‐VCH Verlag GmbH & Co. KGaA, Weinheim. d) Measurement of P3HT electrochemical transistors. Device geometry for OECT testing, with a platinum counter electrode (C.E.) and an Ag/AgCl reference electrode (R.E.). e) Chemical structures of the anions of the potassium salts [fluoride, chloride, bromide, perchlorate, hexafluorophosphate, and bis(trifluoromethanesulfonyl)imide from left to right, respectively]. f) Table of apparent dopant masses during oxidation of P3HT films in various potassium electrolytes g) Characteristic transfer curves (*V*_D_ = −200 mV) with six different electrolytes showing increased transistor current for bulkier anions. Reprinted with permission.^[^ ^20^ ^]^ Copyright 2018, American Chemical Society.

Ion structure and concentration in the electrolyte can alter μC* and the swelling process, highlighting electrolyte composition as a key factor in OMIEC optimization.^[^ ^20^, ^22^, ^77^ ^]^ In polymers such as p(g2T‐TT), increased aqueous electrolyte concentration intensified water uptake, leading to increased lamellar expansion and reduced *μ*. The results underscored the broad influence of electrolyte composition on the interplay between *μ* and C*.^[^ ^21^ ^]^ The degree of swelling was directly linked to reduced C* and demonstrated that the sensitivity of glycolated sidechain polymers is correlated with electrolyte composition. Complementing these findings, Cendra et al. demonstrated that anions of varying sizes and hydration levels influenced p(g2T‐TT) mixed conduction behavior (**Figure** **4**a). Their findings also revealed anion related effects.^[^ ^22^ ^]^ In general, the migration and incorporation of larger or less hydrated anions into the polymer film contributed to larger structural expansion and higher transconductance (Figure 4b,c). In addition, Flagg et al. found that in a typical p‐channel OMIEC, P3HT (Figure 4d,e), smaller and slightly polarizable anions bring in ≈10 water molecules as a solvation shell into the polymer matrix, whereas larger and highly polarizable molecular anions are able to enter the film with less water (Figure 4f).^[^ ^20^ ^]^ As a result, different activation energies were observed for the doping process, which is represented as a lower threshold voltage, greater S–D current, and faster doping kinetics with larger and highly polarized molecular anions (Figure 4g).^[^ ^20^ ^]^

The identity and spatial distribution of electrolyte ions not only influence doping levels and swelling behavior but also modulate the local structure and packing distance within the polymer thin film.^[^ ^20^, ^22^, ^53^ ^]^ While experimental studies have uncovered clear trends on how ion size, hydration shell, or electrolyte concentration influence the degree of swelling, thin film morphology, and *μ*, these measurements often cannot resolve the molecular‐scale mechanisms responsible for these effects. To provide a molecular level explanation, several simulation studies have examined how electrolyte composition governs OMIEC behavior. Matta et al. used MD simulations to explore cation and anion effects in p(g2T‐TT), revealing that cation coordination strength increases with ion radius and charge density and that hydrophobic anions such as TFSI^−^ and ClO_4_^−^ preferentially accumulate at the polymer–electrolyte interface and enhance chelation by increasing surface accessibility of sidechain oxygens.^[^ ^26^ ^]^ MD simulations using the Martini force field showed that reduced hydration levels in poly(3,4‐ethylenedioxythiophene) doped with molecular tosylate (PEDOT:Tos) films lead to densification, lower solvent‐accessible surface area, and a three‐order‐of‐magnitude decrease in Na^+^ and Cl^−^ diffusivity, with increased ion confinement at the polymer surface.^[^ ^78^ ^]^ The coarse‐grained results were compared with all‐atom MD simulation, experimental data, and DFT calculations for validation.

In a system doped with different ions, MD simulations revealed that larger and more hydrophobic ionic liquid anions such as tetracyanoborate (TCB^−^) and tricyanomethanide (TCM^−^) induce microphase separation in PEDOT:PSS by driving PSS segregation and PEDOT aggregation, ultimately improving *π*–*π* stacking and enhancing electronic conductivity.^[^ ^27^ ^]^ A liquid crystalline small‐molecule OMIEC composed of a quaterthiophene (4T) conjugated core flanked by ionically conductive oligoethylene glycol (PEO4) side blocks was also studied using MD.^[^ ^79^ ^]^ In the 4T/PEO4 systems, it was found that salt doping drives a sequence of morphological transformations: from smectic order at low concentrations, to a more interconnected 3D structure with enhanced electronic delocalization at intermediate salt concentration levels, followed by amorphization and suppressed transport under excessive salt loading.

Extending these structural insights into the electronic domain, a multiscale modeling workflow that couples MD with quantum mechanical/molecular mechanical simulations was used to study the mixed conduction properties of p(g2T‐T).^[^ ^80^ ^]^ The simulation revealed that charge localization and redistribution depend sensitively on the spatial arrangement of mobile ions, particularly Cl^−^ clustering, and are dynamically modulated by hydration‐mediated dielectric changes. This hybrid approach updates the electronic charge distribution based on the local ionic and solvation environment extracted from MD, allowing the electronic structure to reflect condition‐specific electrostatic influences.

Together, these results underscore that electrolyte composition and spatial distribution of counterions play a critical role in governing ion penetration, swelling behavior, and the balance between ionic and electronic conduction.

### 6.2 The Role of Dissolved Oxygen and Long‐Term Operational Stability

Among the various environmental factors, dissolved oxygen has been identified as a primary antagonist to OMIEC stability, playing a pernicious dual role. On one hand, oxygen can act as an uncontrolled p‐dopant for many p‐channel OMIECs, particularly those with shallow HOMO energy levels. This unintentional doping can fill electronic trap states, which can paradoxically appear to enhance performance in short‐term measurements.^[^ ^81^, ^82^ ^]^

On the other hand, this same reactivity makes oxygen a potent agent of degradation. A critical degradation mechanism, especially for p‐channel thiophene‐based OECTs, is the oxygen reduction reaction that occurs at the electrode–OMIEC interface.^[^ ^82^ ^]^ This process is most pronounced at the drain electrode when it is under a reductive bias stress. Here, catalytic electrode materials like gold (Au) can facilitate the electrochemical reduction of dissolved O_2_ to form highly reactive oxygen species, such as hydrogen peroxide (H_2_O_2_) or hydroxide ions (OH^−^).^[^ ^83^ ^]^ These mobile, aggressive species can then diffuse into the OMIEC channel, where they chemically attack and degrade the conjugated polymer, destroying its electronic structure and disrupting charge transport.^[^ ^82^ ^]^ This mechanism explains the common observation of accelerated degradation under simultaneous oxidative (at the gate) and reductive (at the drain) bias stress. Electron transport (n‐channel) OMIECs are even more vulnerable, as their electron‐transporting nature makes them intrinsically susceptible to oxidation, and their stability in the presence of ambient oxygen and water is notoriously poor.^[^ ^81^ ^]^

## 7 Conclusions and Outlook

Key studies have underscored sidechain engineering as a critical optimization strategy for achieving desired material performance in OMIECs, with significant conclusions summarized accordingly. Concurrently, the influence of electrolyte composition has also been addressed. Building upon these findings, potential avenues for future research have been outlined in the outlook section.

### 7.1 Conclusions

Achieving a balance between *μ* and C* presents significant challenges in material design. Employing low‐hydration functionalities can address issues of swelling, but this approach might compromise C*. Specifically, research indicates that modifying ethylene glycol sidechain parameters such as length, linkage site, and density can affect the equilibrium between ionic conduction and electronic conduction. These modifications also alter swelling characteristics and tune both the magnitude and selectivity of ion uptake. Furthermore, adjusting the length of an alkyl spacer has proven effective in mitigating the impact of functional groups on the overall mixed conduction properties of the material. Additionally, hybrid designs that incorporate ionic moieties into the sidechain have been explored. Such ionic sidechains facilitate efficient ion exchange while concurrently suppressing excessive water uptake. This approach leads to improved crystallinity and reduced morphological deformation during device operation. Consequently, integrating ionic functionalities in sidechain molecular design represents a promising strategy to decouple hydration from destabilizing swelling to achieve materials with enhanced reliability in aqueous electrolyte systems.

Simultaneously, research to date has demonstrated the influence of electrolyte concentration and anion characteristics on polymer swelling and doping efficiency; however, most data were collected under static or steady‐state conditions. For example, larger anions with weak hydration shells increase *μ* but induce irreversible structural changes, suggesting the need for further exploration under real‐time cycling. Similarly, water uptake modulates doping reversibility and ion transport, yet the implications of repeated environmental shifts (such as pH fluctuations, multivalent ion exposure, and formation of by‐products) on long‐term stability are currently insufficiently addressed.

A critical challenge that remains is ensuring the long‐term operational stability of OMIECs. Recent work has begun to shed light on specific degradation pathways. For instance, in common p‐channel thiophene‐based OECTs, degradation has been linked to the presence of dissolved oxygen in the electrolyte, which can react at the gold drain electrode under reductive bias. This process generates reactive oxygen species that chemically damage the conjugated polymer, leading to a decline in performance. The results highlight that device failure is not solely a property of the OMIEC material itself but involves a complex interplay between the polymer, electrolyte, and electrode materials. Therefore, strategies to enhance stability must be multifaceted, involving not just polymer design but also device engineering.

### 7.2 Future Outlook

While current modifications to ethylene glycol sidechains using alkyl spacers show promise, these findings often result from case‐by‐case analyses. Consequently, a significant increase in research dedicated to establishing robust quantitative models is anticipated. Leveraging advanced in situ and *opera*n*do* characterization techniques with advanced simulations, it is anticipated that quantitative models can be developed to precisely correlate diverse sidechain molecular design parameters (including spacer length, number of ethylene glycol units, branching, density, and specific linkage points) with critical material properties such as ionic/electronic conductivity, ion selectivity, and the degree of swelling.

Second, research efforts to date have focused predominantly on experimental characterization of how specific changes in molecular structure impact resultant polymer mixed conduction properties. This largely phenomenological approach leads to a notable gap in elucidating the underlying mechanisms responsible for these changes. Thus, future investigations should meticulously examine factors such as electrostatic interactions, Donnan exclusion, and selective ion uptake. Furthermore, establishing a quantitative analytical framework is crucial for analyzing ion–dipole interactions and the solvation capabilities of other common polar functional groups. Such a methodology would be instrumental in determining whether these alternative groups offer solvation comparable to ethylene glycol units and in understanding how they modulate key transport parameters, including ion mobility, volumetric charge capacitance, and electronic charge carrier mobility.

Third, addressing the stability issues is paramount for the practical application of OMIEC‐based OECT devices. Future research should systematically investigate degradation mechanisms under a wider range of operating conditions and electrolyte compositions. This includes exploring strategies to mitigate degradation, such as the removal of dissolved oxygen from the electrolyte, replacing traditional gold electrodes with more stable materials, or developing passivation layers to protect the electrodes. Alternatively, two‐step strategies (e.g., one that combines solvent degassing with controlled chemical predoping) may be promising future avenues to improve stability. A deeper, mechanistic understanding of the dynamic interplay between the OMIEC, ions, solvent, and electrodes is essential for designing next‐generation materials with inherent long‐term stability.

Fourth, building upon initial successes associated with incorporation of ionic sidechain groups, the scope for conjugated polymer molecular design has been expanded to include weakly dissociating ionic functional groups, such as amines (—NH_2_) or carboxylic acids (—COOH). The integration of such weakly dissociating units offers an additional mechanism by which OMIEC response can be modulated. Specifically, the degree of dissociation may be expected to regulate hydration levels and ion uptake. New trends toward the design of multifunctional sidechains employing hybrid strategies that incorporate various ionic moieties are anticipated. Such designs could synergistically combine optimized segments for ion transport, precisely engineered spacers, and strategically positioned novel ionic groups to achieve heretofore unprecedented control over water uptake and morphology. Combining these approaches will lead to the design, development, and implementation of precisely engineered molecular structures to achieve application specific desired outputs.

## Conflict of Interest

The authors declare no conflict of interest.
